# Unveiling urban marathon development characteristics and urban growth strategies in China: Insights from time series analysis of Baidu Search Index

**DOI:** 10.1371/journal.pone.0287760

**Published:** 2023-06-30

**Authors:** Erchang Zheng, Chengbin Xue, Gongqiang Chen, Yinghui Zhang, Jinchuan Zou

**Affiliations:** 1 Sports Institute, Huaqiao University, Quanzhou, China; 2 School of Management, Yang-En University, Quanzhou, China; Queen Mary University of London, UNITED KINGDOM

## Abstract

The strategic exploration of urban sports tourism resources and the pursuit of novel trajectories for urban growth are pivotal for resource integration and competitive enhancement within cities. This investigation concentrates on Chinese city marathons and compiles daily search index data from Baidu for 38 city marathons across the nation, spanning from January 1st, 2012 to May 3rd, 2022. Employing time series clustering to evaluate the data, and in conjunction with indices related to urban tourism resources and city development, we delve into the characteristics of how Chinese city marathons propel urban growth. The findings illustrate that the search index data for the 38 city marathons can be clustered into three categories, with Xi’an, Fuzhou, and Dalian emerging as the epicenters of clustering. The representative search index data for these three clusters reveal diverse characteristics of change. The search index shifts for three landmark races align generally with the changes observed in their respective cluster center races, however, variations exist among the search index changes for these iconic marathons. The degree of search index and its trending direction in city marathons emanate from the synergistic influence of the city’s political, economic, and tourism attributes, in addition to the event’s prominence. City marathons also catalyze urban development through economic stimulation, image enhancement, and infrastructure improvement. Future exploration of novel trajectories for urban development could be facilitated through harnessing the economic and tourism attributes of these events, and by orchestrating a unified series of marathons.

## 1. Introduction

In the modern era, marked by rapid globalization and economic shifts, cities around the globe have experienced a surge in the free flow of diverse resources. This has prompted many cities to strive for resource integration, overall competitiveness enhancement, and economic transformation as new avenues for development. In this context, exploiting urban tourism and sports resources to construct an integrated development path has emerged as an innovative direction. Particularly, sports events and venues, serving as vital components of urban sports resources, play a pivotal role. Among them, marathon events, owing to their wide reach, high participation, and significant influence, present an intriguing blend of sports and tourism characteristics. Focusing on the developmental traits of Chinese city marathons symbolizes a step forward in understanding the development characteristics and optimization paths of Chinese sports tourism, thereby opening new avenues for urban development models. Time series, a prevalent form of high-dimensional data associated with time, such as stock data [1, 2], financial data [3, 4], has grown dramatically with the progression of social economics and information technology. Leveraging data mining technology to scrutinize the popularity time series data of Chinese city marathons stands as a compelling method to uncover potential useful insights in marathon events.

Our research motivation can be categorized into three distinct facets. Firstly, the city-specific attributes of marathon events can serve as valuable benchmarks for other cities to emulate successful experiences. The incorporation of clustering results with political, economic, and tourism factors of varied cities allows for an in-depth extraction of city characteristics associated with marathon events. Secondly, the sustenance of high event popularity emerges as an essential prerequisite for events to boost urban development. The popularity trends during representative marathon event seasons can be compared to investigate the correlation between event intensity and popularity. Lastly, it is of paramount importance to discern the drawbacks of the prevailing city development path steered by marathon events. We first delineate the existing paths and subsequently propose innovative developmental paths.

A comprehensive classification of 38 city marathon events into three categories, with Xi’an, Fuzhou, and Dalian as the clustering centers, reveals distinct oscillation patterns of the representative season popularity in various categories. Analysis from the perspectives of politics, economy, and tourism, illustrates that city marathon events predominantly propel urban development through pathways such as economic growth stimulation, city image enhancement, and urban infrastructure facilitation.

We summarize our contributions as follows:

- By implementing data mining techniques, we measure and cluster the popularity data of 38 city marathon events, broadening the research subjects and methodologies in this field.
- We examine the 10-year popularity data of 38 city marathon events from both longitudinal and latitudinal perspectives, amplifying the theoretical accomplishments of this research domain on the city marathon events’ impact on urban development.
- Capitalizing on the clustering outcomes, we extract the characteristics of different marathon event cities from the perspectives of city politics, economy, and tourism. By blending the existing development paths driven by city marathon events, we suggest novel enhancement paths, providing experiential references for cities with analogous characteristics.

## 2. Related work

The emergence of sports tourism has been instrumental in advancing cities in terms of economic growth, mobility resource attraction, urban image enhancement, and urban competitiveness bolstering. The successful model of sports tourism in urban development provides feasible alternatives for other city development paths. Existing research primarily focuses on the evaluation of sports tourism resources and city development path that is contingent on sports tourism value. This evaluation entails a comprehensive assessment of sports tourism resources from various angles including strengths, weaknesses, opportunities, challenges, etc. [5, 6], encompassing sports resources, ecological resources [7, 8], tourism resources [9, 10], and geographic resources [11, 12]. Based on these evaluations, further explorations are conducted to identify the development path of urban sports tourism, integrating local resources and regional attributes to propose a fitting development path for sports tourism in the region, thus driving regional integration [13, 14]. The employed research methodologies primarily consist of theory-based qualitative research methods. However, with the continuous refinement of statistical methods, an increasing number of researchers are integrating mathematical statistics and data mining methods into their research. The theoretical research chiefly leverages theories and methods such as SWOT analysis [15], RMP theory [7], and logical research to explore local sports tourism development models. Quantitative research typically employs conventional statistical methods such as questionnaire surveys and mathematical statistics [16, 17], with data mining methods gradually making their way into this field [18]. The combination of qualitative and quantitative methods to explore the development path of urban sports tourism has become a novel direction for city development.

Marathons, as representative events of sports tourism, serve as a significant avenue to explore new models of sports tourism and urban development. Research concerning urban marathons and urban development mainly concentrates on three aspects: Motivation for Sports Tourism, the factors influencing tourist satisfaction, and the paths through which marathons stimulate high-quality urban development. Current motivations for participating in marathon events primarily consist of fitness motivation [19], emotional satisfaction [20, 21], social motivation, consumption experience [22], and destination attraction [23]. The study of factors influencing tourist satisfaction is conducted from the perspectives of marketing, management, and participants. From a marketing perspective, significant factors influencing participant satisfaction include marathon event product design, safety, advertising, and promotional materials [24–26]. From a management perspective, the organizational and operational mechanisms, the level of operational management, and the incorporation of market mechanisms directly influence the success of the marathon event [27]. From the perspective of participant involvement, the fusion of Internet of Things and other technologies with marathons can significantly enhance participant involvement and satisfaction [28]. Research on the path of marathons contributing to high-quality urban development primarily commences from two angles: economic and cultural. It analyzes the positive effects of marathons on the city’s public fitness, economic development, cultural heritage, and brand development [29]. Drawing from the development background, organizational model, and operation mechanism of urban marathons [30], this research outlines its development characteristics and limitations, and then suggests applicable paths for marathons and high-quality urban development. A relatively novel perspective proposes the development of sustainable sports tourism, advocating that environmental protection should be considered one of the goals in promoting urban development through sports tourism. Destination planning and community engagement are regarded as crucial strategies to achieve this objective [31, 32].

In light of previous research, sports tourism and urban development have emerged as a new research focus, yielding abundant research outcomes. Evaluating urban sports tourism resources and exploring the sports tourism development path are vital research topics. Qualitative analysis remains the primary method, but quantitative analysis is gaining traction. Urban marathons stimulating urban development is an important research trajectory. Nonetheless, certain issues persist: (1) the majority of qualitative and quantitative analyses are grounded in theories and questionnaire data, which may introduce a degree of subjectivity to the research findings; (2) research methodologies mainly rely on theoretical analysis and conventional statistical methods, lacking in-depth data analysis; (3) research subjects primarily focus on individual city sports tourism, lacking a comparative analysis of national events. In response to these issues, this research utilizes marathon events as the research object, collects Baidu search index data of 38 urban marathons nationwide over 10 years, employs data mining methods to analyze the data, and combines relevant indicators of urban development to explore the characteristics and improvement paths of Chinese urban marathons in driving urban development.

## 3. Study design and data analysis

**Fig 1:**
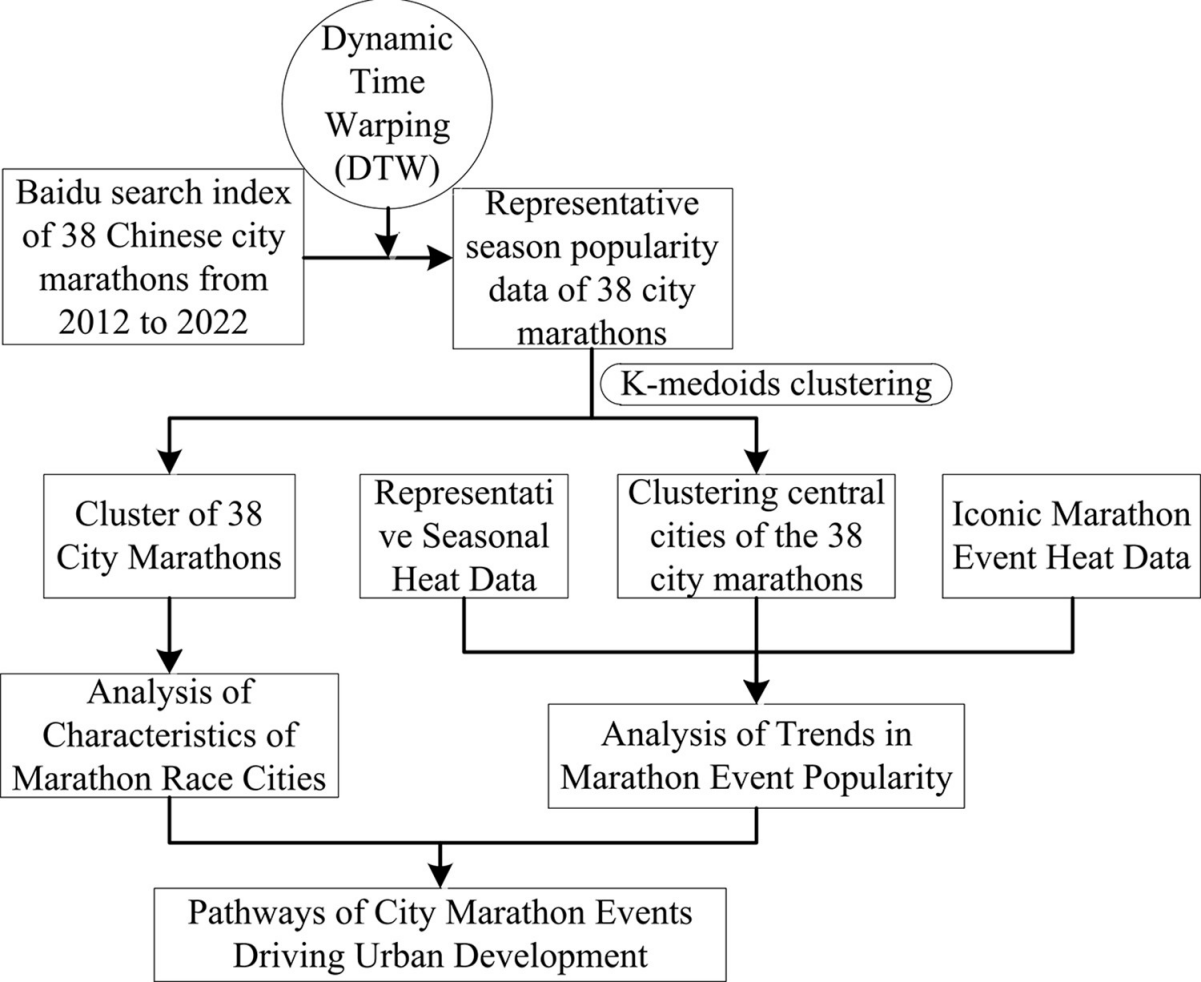
Research design process for the development characteristics of Chinese urban marathons.

This research utilized data science methods, such as similarity measurement and clustering, to analyze data from 38 Chinese city marathons. The marathons were categorized into three clusters based on their popularity, and three representative cities were delineated for each cluster. The characteristics of the marathon cities within distinct clusters were examined in concert with other urban development variables. Utilizing the clustering centers and data on hallmark seasons and landmark events, trends and patterns of marathon popularity within each cluster were summarized. Upon scrutinizing the data and considering the urban development factors, a path for urban development via city marathons was suggested (Fig 1).

### 3.1 Data source

In this investigation, the researchers targeted 66 Chinese city marathons that were certified by the International Association of Athletics Federations (IAAF) as of April 2022. These marathons were stratified into three tiers: Marathon International Platinum Label, International Gold Label, and International Label. The Baidu Search Index, which signifies the frequency with which a keyword is employed in a specific field, was utilized as a metric of the marathons’ popularity. The Baidu Search Index was selected due to its representativeness and authority, considering that Baidu Search commanded 85.48% of the market share of search engines in China in 2021.

Policy factors triggered the surge of marathon events in China beginning around 2015. As of 2022, all the marathon events in China that were endorsed by the International Association of Athletics Federations (IAAF) were established prior to 2018. Nonetheless, preceding 2015, some marathon events had already attained a certain degree of development, and data from this period retains substantial research relevance. Therefore, marking 2015 as the midpoint, the data collection for this study commenced three years earlier, in 2012, and spanned until 2022, delineating the data collection timeframe (Table 1).

**Table 1:** Inaugural edition dates of marathon events in 38 cities.

No.	city	time	No.	city	time	No.	city	time
1	Shanghai	1996.09.28	14	Xianggang	2015.01.21	27	Changsha	2015.10.18
2	Xiamen	2003.11.27	15	Dongying	2013.05.12	28	Shenyang	2015.09.27
3	Guangzhou	2012.11.18	16	Beijing	1981.09.27	29	Hengshui	2012.09.22
4	Shenzhen	2013.12.08	17	Changzhou	2017.10.14	30	Taiyuan	2010.09.05
5	Kunming	2016.12.17	18	Liupanshui	2013.08.10	31	Haerbin	2016.08.28
6	Hefei	2014.11.16	19	Jilin	2016.06.26	32	Lanzhou	2011.07.03
7	Nanchang	2016.11.20	20	Dalian	1987.05.17	33	Guiyang	2014.06.29
8	Hangzhou	1987.10.11	21	Xian	2017.10.28	34	Chongqing	2011.03.19
9	Xichang	2014.11.09	22	Yangzhou	2006.05.27	35	Xuzhou	2017.04.09
10	Wuhan	2016.04.10	23	Wuxi	2014.04.13	36	Yichang	2016.10.23
11	Nanjing	2015.11.29	24	Fuzhou	2015.12.20	37	Zheng Kai	2007.05.06
12	Chengdu	2017.09.23	25	Nanning	2016.12.04	38	Qingyuan	2015.03.15
13	Suzhou	2017.12.16	26	Shaoxing	2017.11.11			

To ascertain the Baidu Search Index for each marathon, the researchers arranged and amalgamated the words in the names of the 66 city marathons and searched for them individually in the Baidu Search Index search interface. They discovered that 39 cities corresponding to 38 city marathons had established keywords in the Baidu Search Index database. Consequently, the keyword data for these 38 city marathon events was selected as the final research subject.

This study provides a comprehensive analysis of the popularity trends of 38 city marathons in China that are certified by the IAAF. The Baidu Search Index served as the popularity indicator, with data collected from January 1, 2012, to May 3, 2022, encompassing the search index for PC and mobile terminals as well as an information index launched in July 2017. The data for each city marathon is presented in Table 2.

**Table 2:** Fields of Baidu search index data for city marathon events.

Date	Event name: Shanghai Marathon			
	search index(all)	search index(pc)	search index(wise)	information index
2012-01-01	78	78	0	-
2012-01-02	122	64	58	-
2012-01-03	128	68	60	-
⋮	⋮	⋮	⋮	⋮
2022-05-03	154	70	84	0

### 3.2 Descriptive statistical analysis

Table 3 discloses the descriptive statistics of the dataset. A notable observation from the table is that the median values of marathon event popularity data across each city are considerably lower than their respective mean values, whereas the standard deviations are significantly larger than the means. This is indicative of a skewed distribution favoring extreme values and a relatively dispersed distribution. This is primarily attributed to the fact that during non-event seasons, the event popularity typically dwindles or even vanishes. However, with each event announcement during event seasons, the popularity momentarily surges. To counteract potential analytical inaccuracies triggered by such fluctuation, we implement a slicing technique on the event popularity data guided by the annual registration and event dates. A detailed exposition on the specific slicing method will be covered in the similarity calculation section of this study.

**Table 3:** Descriptive statistics of the data objects.

Host city	Minimum value	Average	Median	Standard deviation	Variance	Maximum value
Xiamen	63	591.98	271.5	1635.72	2675564.45	56329
Shanghai	64	584.57	326	1245.58	1551470.24	30840
Shaoxing	0	96.48	0	259.18	67176.39	7453
Shenzhen	0	353.74	188	760.91	578979.12	21050
Shenyang	0	152.96	126	432.28	186869.33	13906
Beijing	0	643.12	312	1934.51	3742332.02	68701
Changzhou	0	6.54	0	74.89	5608.82	4307
Chengdu	0	204.39	147	503.32	253335.79	15271
Dalian	0	205.09	143	360.99	130313.10	9357
Dongying	0	133.51	123	239.17	57204.20	6924
Fuzhou	0	122.58	64	274.46	75329.63	6474
Guangzhou	0	496.36	243	970.41	941692.56	22182
Guiyang	0	100.54	63	182.40	33268.08	3868
Haerbin	0	176.11	68	1657.13	2746073.78	93118
Hangzhou	0	453.06	217	859.36	738491.16	16564
Hefei	0	157.85	137	270.22	73019.64	7245
Hengshui	0	93.32	63	124.90	15599.67	2590
Jilin	0	67.30	0	242.56	58833.66	10832
Kunming	0	104.51	66	387.05	149806.83	15658
Lanzhou	0	297.44	157	993.12	986289.19	39525
Liupanshui	0	120.16	69	257.40	66257.22	6614
Nanchang	0	109.47	63	286.20	81910.43	11489
Nanjing	0	268.72	153	726.25	527444.93	22848
Nanning	0	99.57	64	150.25	22575.51	2302
Qingyuan	0	95.35	61	202.89	41166.13	5316
Suzhou	0	161.24	133	269.60	72685.08	10937
Taiyuan	0	183.19	135	300.68	90409.14	7812
Wuxi	0	348.61	176	784.49	615429.69	19885
Wuhan	0	341.57	174.5	1363.13	1858136.58	66142
Xi’an	0	230.82	133	889.89	791904.89	28677
Xichang	0	83.28	59	425.09	180699.11	19751
Hong kong	0	153.93	139	181.86	33071.25	6000
Xuzhou	0	116.58	63	296.22	87747.37	8403
Yangzhou	0	239.75	151	406.07	164894.26	7809
Yichang	0	65.51	0	148.56	22070.18	3645
Changsha	0	175.73	135	728.99	531430.86	39060
Zheng Kai	0	381.83	209	606.96	368394.54	13849
Chongqing	0	289.90	177	573.79	329235.74	17021

### 3.3 Selection of representative seasons for marathon events in various cities

#### 3.3.1 Dynamic Time Warping (DTW)

**Fig 2:**
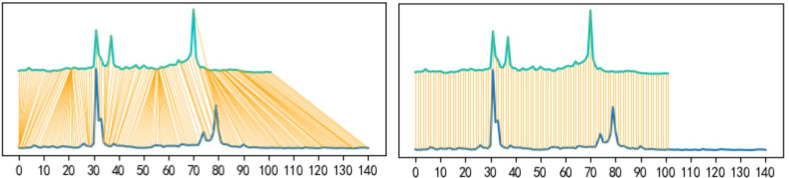
Comparison of Euclidean distance (a) and DTW distance (b) for two seasons of Shanghai marathon.

Dynamic Time Warping (DTW) is a similarity measurement method that employs a warped time axis to better match and map the forms of time series. It measures the relationship between distinct objects. In time series data mining, this similarity measurement is crucial and fundamental. Initially, DTW was applied to process speech data, and subsequently Berndt employed it to measure time series similarity. Unlike traditional similarity measurement, which calculates point-to-point distance only for equal length time series, DTW aligns the most similar points based on the resemblance of the time series shapes when calculating the distance (Fig 2). As such, DTW has found widespread application in the field of time series data mining.

DTW outperforms traditional similarity measurement methods, for it:

1. Can measure similarity not just for time series of equal lengths, but also for those with unequal lengths.
2. Is not sensitive to alterations or anomalies in time series, hence more suitable for measuring such data.
3. Can facilitate asynchronous similarity comparison.

By employing DTW distance measurement, city marathons with comparable or identical popularity trends can be clustered into the same category during subsequent clustering process. This mitigates the influence of differing time lengths and delays of marathon popularity data on the clustering outcomes, and prevents similar marathons from being clustered into different categories due to variations in the time of marathon popularity or popularity lag, resulting in large popularity distances.

#### 3.3.2 Process for selecting representative seasons

**Fig 3:**
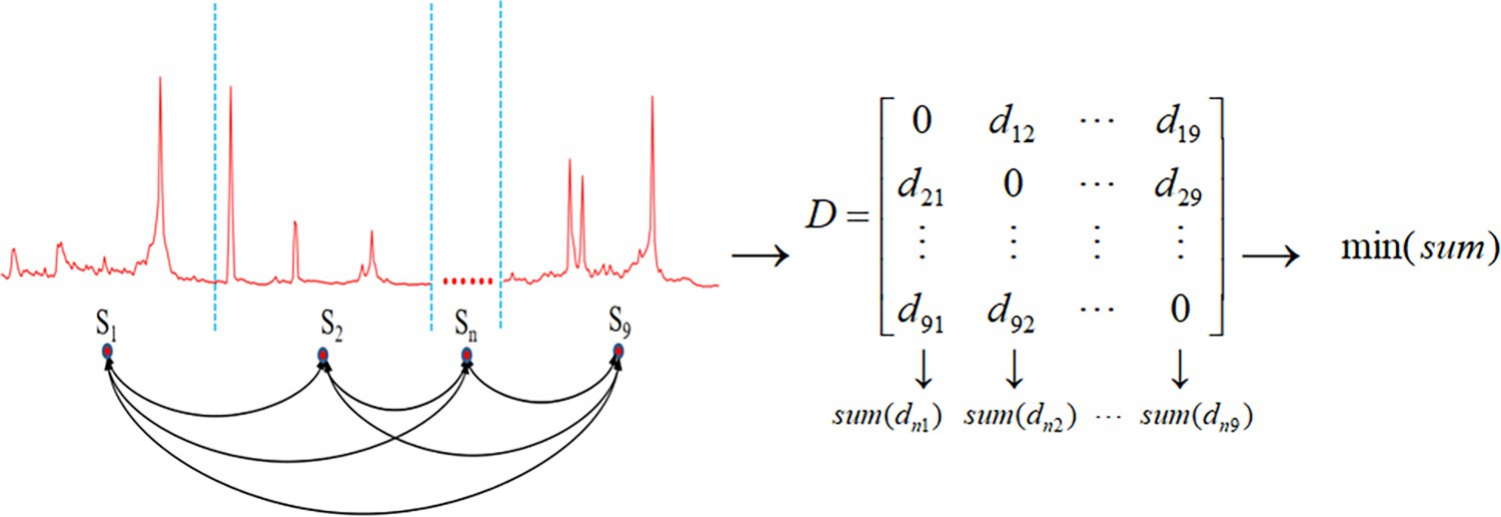
Flowchart of selecting representative season for a certain city’s marathon event.

By combining the start registration time and start time of each marathon, the daily search index of each city’s marathon event was obtained for the 31 days preceding registration and the 31 days following the start time. This data was used to gather data for all seasons of various city marathon events. For each city’s marathon event, the DTW distance was calculated between every pair of seasons, forming a DTW distance matrix between each season of the city’s marathon event. The season with the least cumulative DTW distances with other seasons was selected as the representative season for that city’s marathon event, thereby obtaining the standardized popularity data for each city’s marathon event’s representative season. The representative season selection process is illustrated in Fig 3.

Taking data from the Shanghai Marathon event as an example, S1, S2…, Sn (n = 1, 2…, 9) represent the popularity data of the nine seasons of the Shanghai Marathon event from 2012 to 2022 (the event was not conducted in 2021 due to the epidemic, and the 2022 event has not yet taken place). Each season’s data reflects the daily search index during the 31 days before registration and the 31 days after the start time of that year’s Shanghai Marathon event. From these nine seasons, the season with the most representative popularity trend for the Shanghai Marathon event was selected, this season should possess the smallest cumulative distance with other seasons’ popularity trend sequences. Utilizing the DTW distance measurement method, the distance between the popularity trend data of the nine seasons of the Shanghai Marathon event was calculated, procuring the distance matrix D (Fig 3). The value of d12 in the matrix signifies the DTW distance between the popularity trend sequences of the first and second season of the Shanghai Marathon event. Summing each row or column of the matrix provides the cumulative distance between the popularity trend sequences of each season of the Shanghai Marathon event and the other seasons. The season with the least cumulative distance with other seasons’ popularity trend sequences is considered as the representative sequence for the Shanghai Marathon event.

### 3.4 Cluster analysis

#### 3.4.1 K-medoids algorithm

Clustering represents a standard machine learning technique employed in data mining. Its fundamental aim is to segment sample objects into multiple subsets, known as "clusters," such that objects within the same cluster exhibit maximum similarity, whereas those in different clusters exhibit maximum dissimilarity. The objective function is therefore to maximize or minimize within the same cluster and the converse across different clusters. There exists a vast array of intricate and diverse clustering algorithms, each carrying their unique advantages, disadvantages, and applicable scenarios.

In this research, we adopted the K-medoids algorithm, an offshoot of the popular K-means clustering algorithm, to cluster the popularity data of representative marathon seasons across various cities. PAM (Partitioning Around Medoids) is among the earliest proposed K-center algorithms and a partition-based clustering algorithm notable for its robustness, high accuracy, and its avoidance of classical K-means limitations.

Application of the K-medoids algorithm facilitated obtaining several representative clusters that effectively capture the current trend of marathon popularity alterations from a data mining perspective. This analysis aids in identifying cities with similar marathon popularity data, characterizing marathon event clusters represented by specific events, and summarizing trends of representative events.

#### 3.4.2 Cluster results

**Fig 4:**
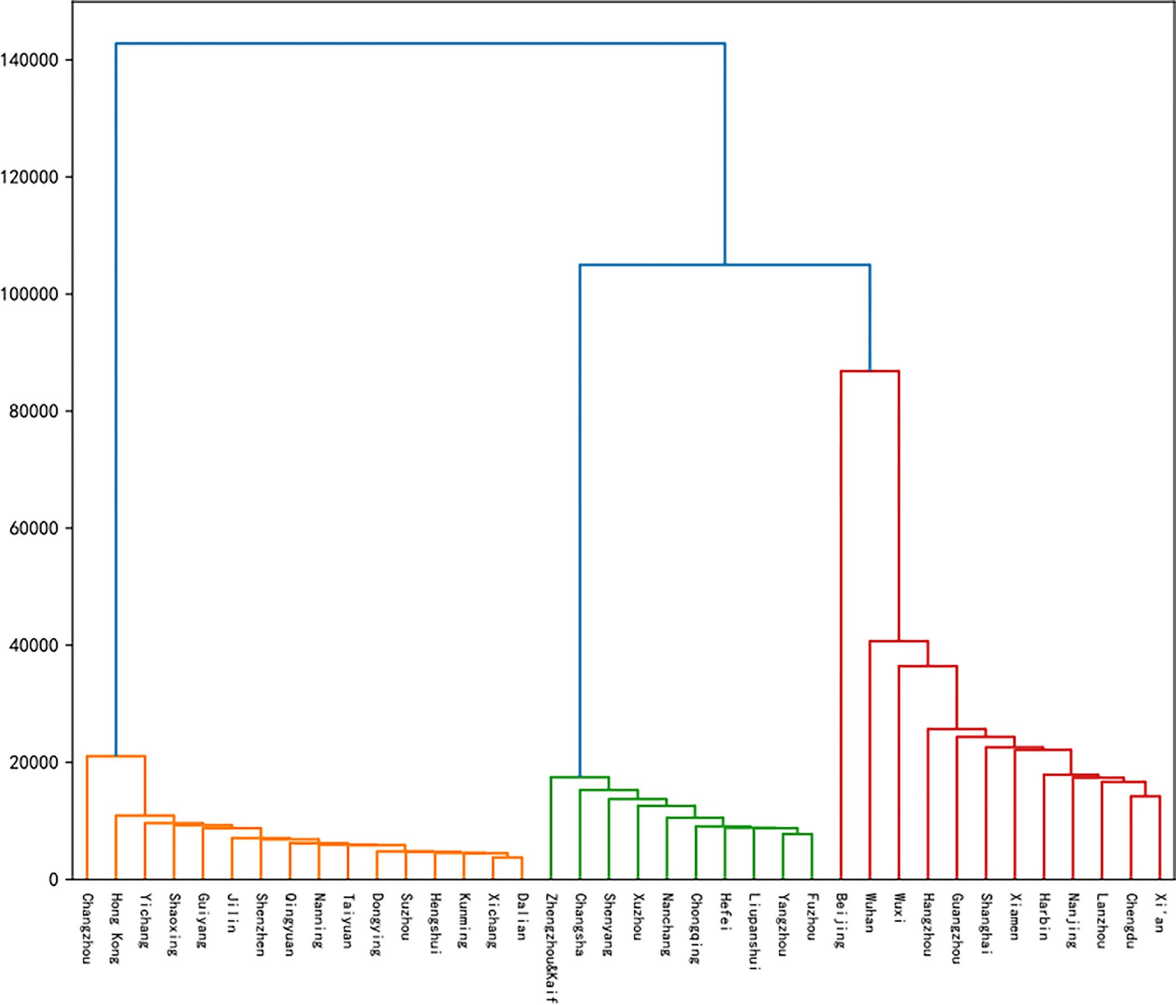
Clustering results of 38 marathon races in cities.

Corresponding to the representative season popularity data for each marathon race, the DTW distance was computed pairwise between representative seasons for each city’s marathon event, generating a DTW distance matrix of representative seasons’ popularity data. This matrix was subsequently input into the K-medoids model with a predetermined k value of 3 to obtain the clustering outcome (Fig 4).

Based on the clustering outcome, 38 city marathon events were segmented according to their city’s trend in popularity sequence data. These were primarily clustered into three clusters with Xi’an, Fuzhou, and Dalian serving as the clustering centers, respectively. The cluster centered on Fuzhou mainly included 9 cities such as Liupanshui, Nanchang, Hefei, Xuzhou, Yangzhou, Shenyang, Zhengzhou-Kaifeng (Zhengzhou and Kaifeng), Chongqing, and Changsha. The cluster centered on Dalian mainly included 15 cities such as Dongying, Nanning, Jilin, Taiyuan, Yichang, Changzhou, Kunming, Shenzhen, Qingyuan, Suzhou, Hengshui, Shaoxing, Xichang, Guiyang, and Hong Kong. Events clustered in the same cluster had similar characteristics of hosting city, promotion methods, and time interval between registration and the start of the race.

**Fig 5:**
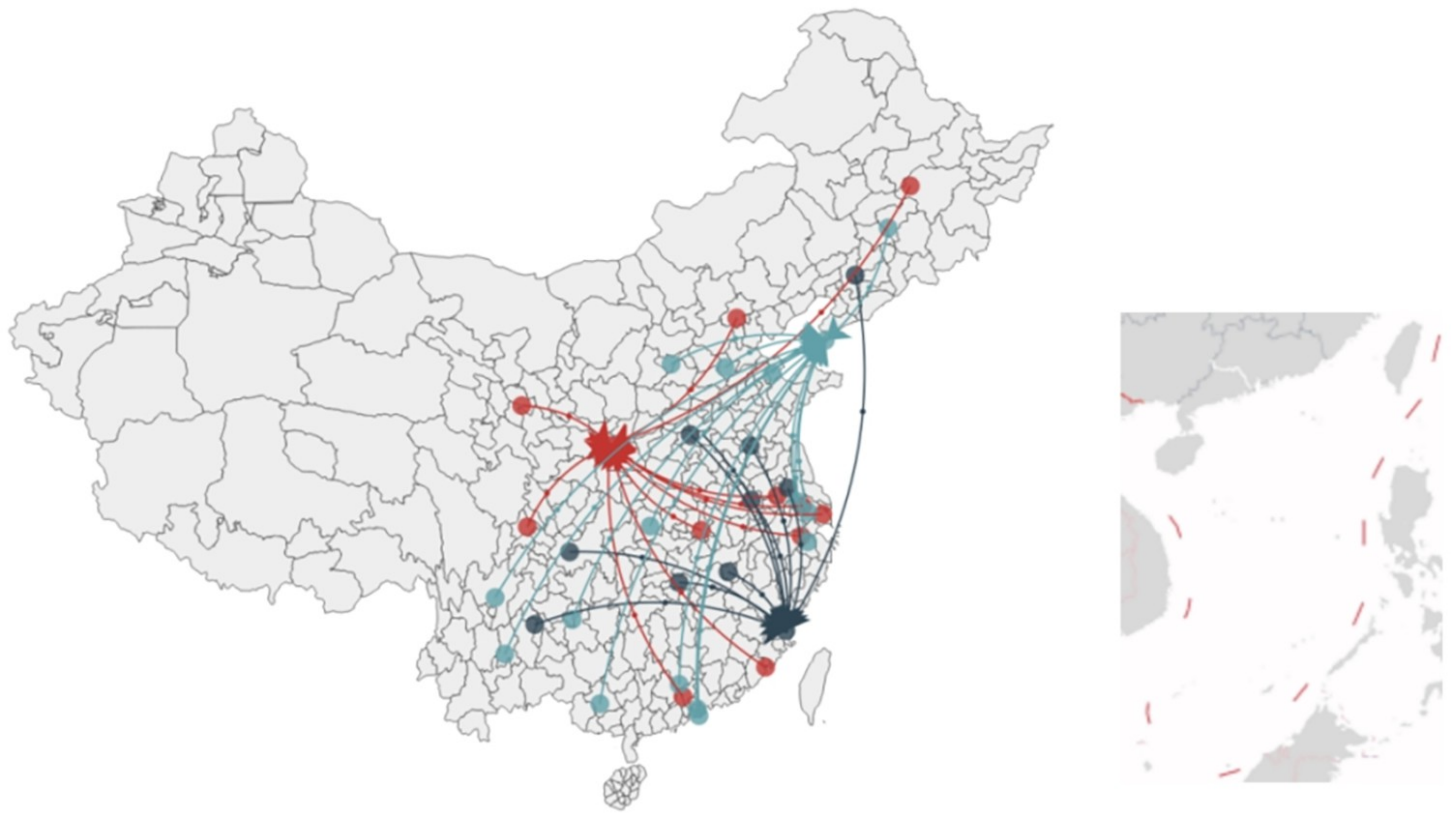
Geographical representation of the clustering results for the heat levels of 38 marathon events in Chinese cities.

As depicted in Fig 5, the most frequented marathons in China are primarily located in the southeastern and central regions, while the northern and northwestern regions host fewer notable marathons. The cluster of marathons centered around the Xi’an Marathon radiates mainly from Xi’an itself. This cluster is characterized by the high popularity of the event and its frequent hosting in provincial capital cities. The Fuzhou Marathon, on the other hand, is the focal point of a cluster of marathons located predominantly in non-coastal, second-tier cities that serve as provincial capitals and harbor unique features. The marathon cluster centered on the Dalian Marathon spans the eastern, southern, and southwestern regions of China. These events are predominantly staged in second-tier cities, their locations shaped by historical factors and the event’s influence. The Shenzhen Marathon also belongs to this cluster. Despite the robust international influence of the Hong Kong Marathon, it does not stand out in the Baidu index due to restrictions on cross-border travel and search engine usage in mainland China. Regions and countries outside mainland China tend to use the Google search engine more frequently, rendering the Baidu index less effective in reflecting the popularity of the Hong Kong Marathon in these areas.

### 3.5 Event heat analysis

The clustering outcomes premised on the popularity of 38 marathon events in China cities have Fuzhou, Dalian, and Xi’an as the clustering centers. The clustering centers’ scientific significance denotes that the representative marathon events from these centers maintain the minimum popularity distance with all other events within the same cluster, embodying the popularity trend for most city marathons within the respective cluster. Consequently, the representative seasonal popularity trends of the clustering center cities, Fuzhou, Dalian, and Xi’an, are utilized as the trend identifiers for their respective categories of marathon events.

As of May 2022, the Shanghai Marathon and Xiamen Marathon stand out as the only two international platinum label marathons endorsed by the International Association of Athletics Federations (IAAF) within China. Beijing, as the capital city, sees its urban appeal propel the popularity of its marathon events above other cities. Thus, the marathon events of Beijing, Shanghai, and Xiamen are selected as flagship events for the analysis of popularity change trends.

The analysis of popularity change trends of these six marathon events assists in investigating the popularity change trends of representative and iconic marathon events across China’s 38 cities, laying the groundwork for subsequent factor analysis and pathway exploration.

**Fig 6:**
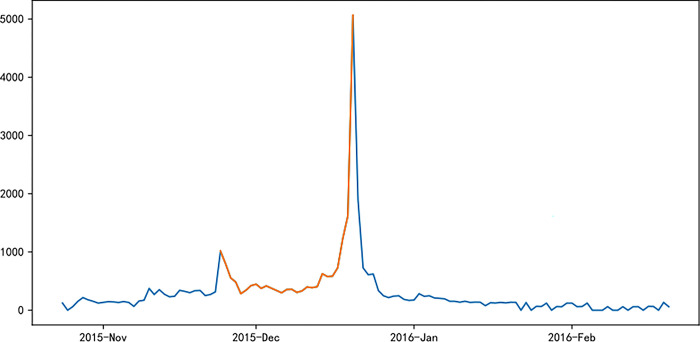
Heat trends of Fuzhou marathon.

**Fig 7:**
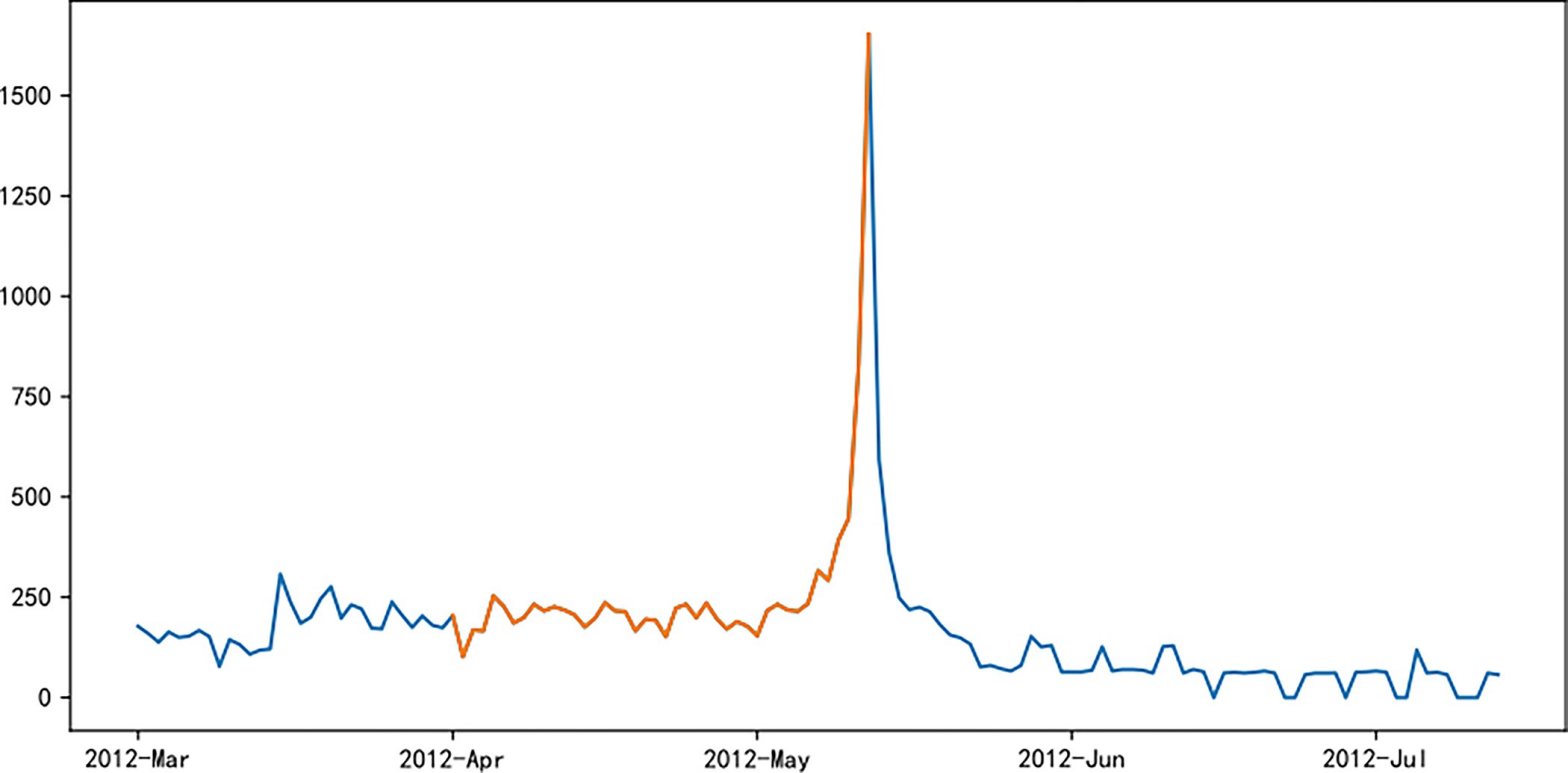
Heat trends of Dalian marathon.

**Fig 8:**
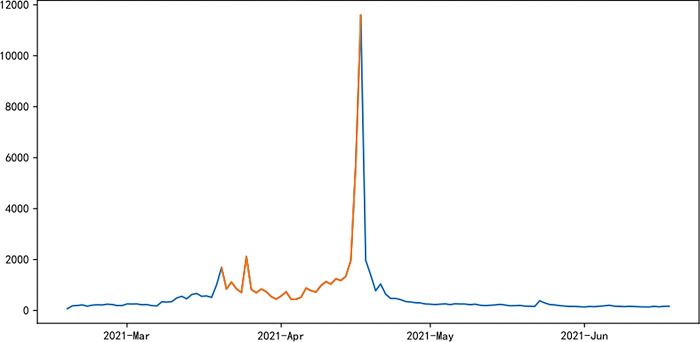
Heat trends of Xi’an marathon.

**Fig 9:**
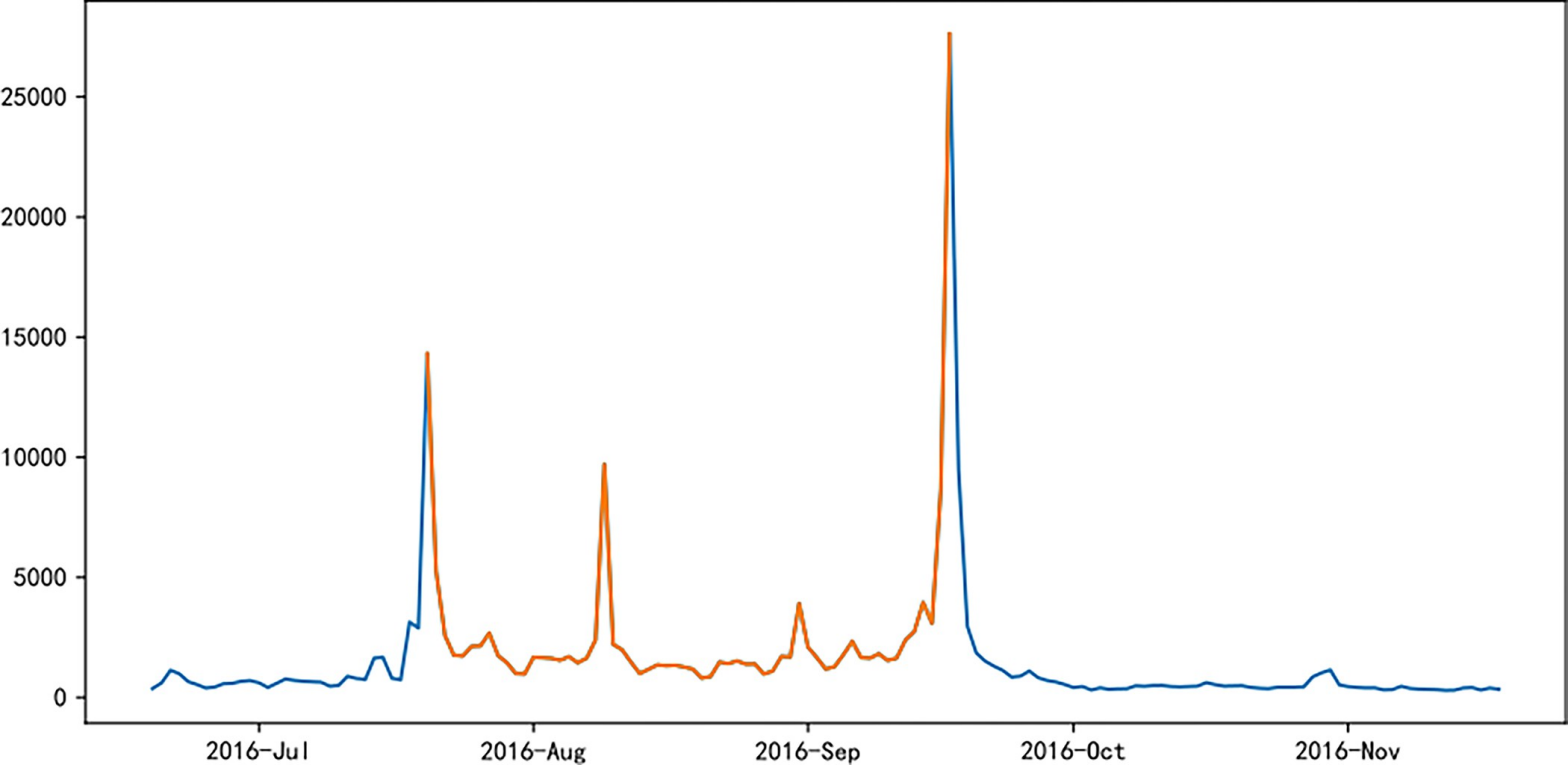
Heat trends of Beijing marathon.

**Fig 10:**
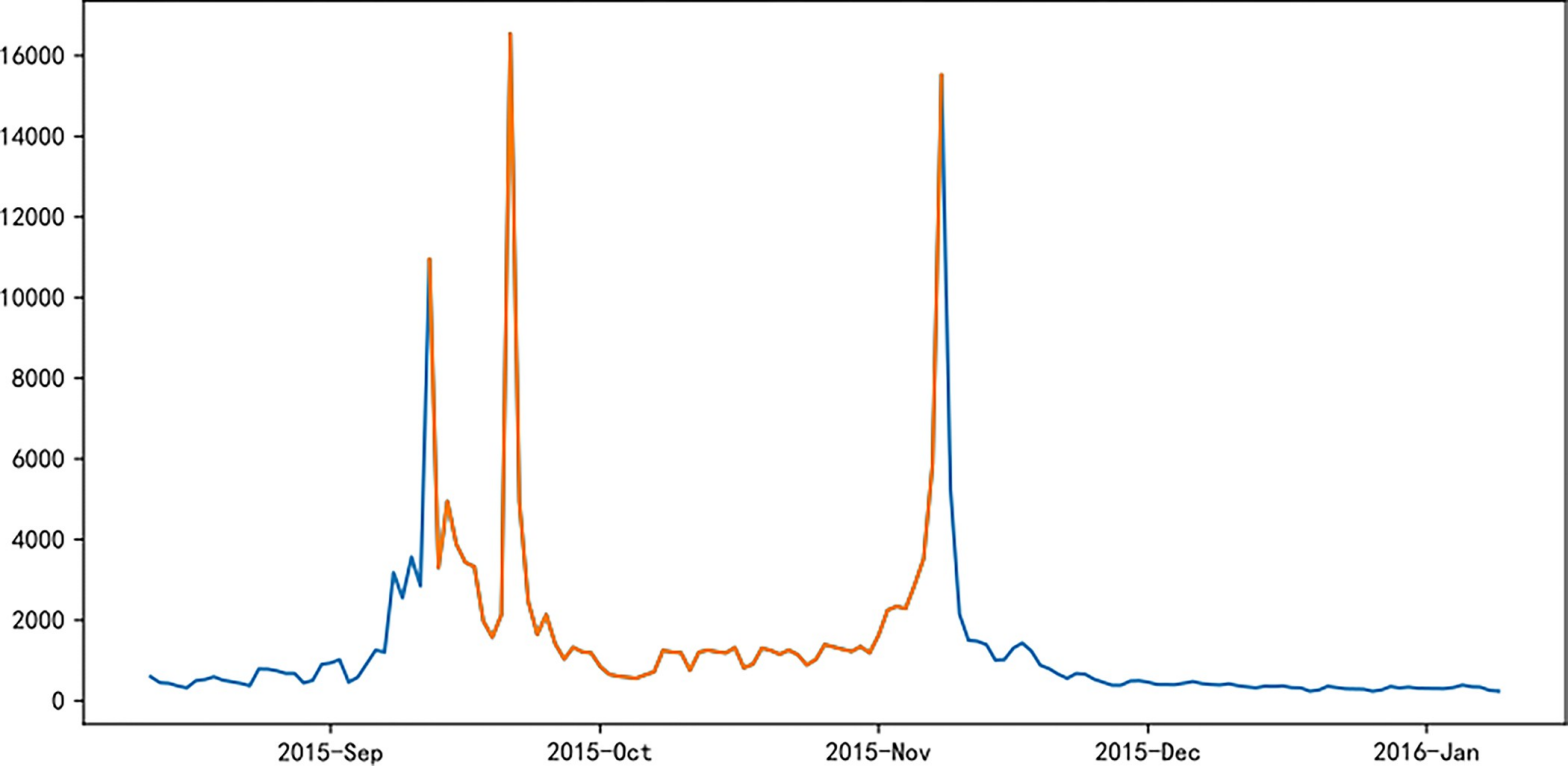
Heat trends of Shanghai marathon.

**Fig 11:**
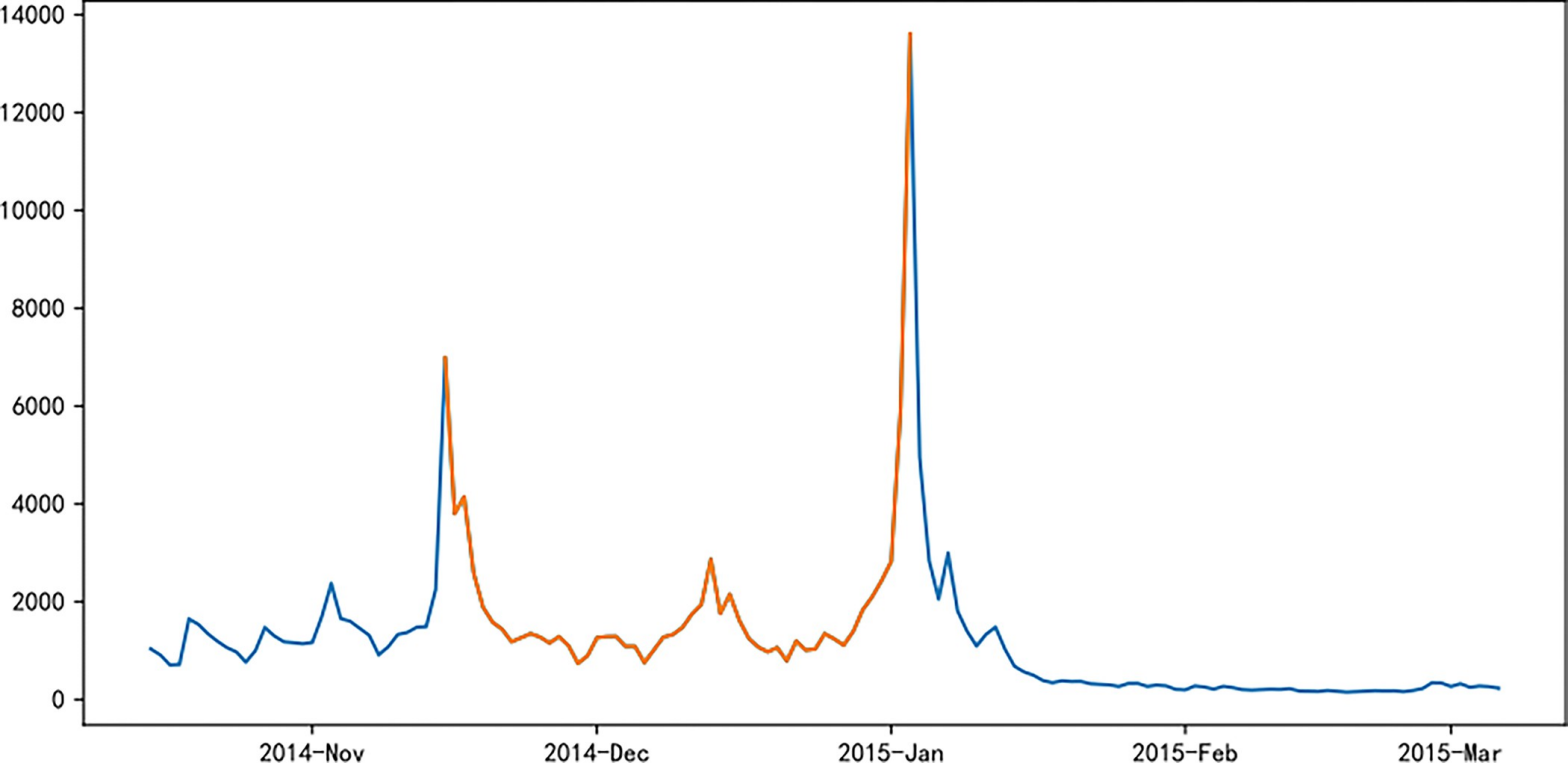
Heat trends of Xiamen marathon.

Figs 6–11 visually displays the seasonal popularity trends signified by the clustering center cities for each of the three clusters. The cluster centering around Fuzhou Marathon illustrates a fluctuating upward trend in popularity about 20 days pre-registration, leading to a minor peak around the registration period and an eventual decrease. However, popularity soon peaks before and after the event before it gradually reverts to baseline levels. Meanwhile, the Dalian Marathon cluster exhibits an upward trend about 20 days pre-registration, maintains at a certain level with minor oscillations leading up to the event, after which the popularity surges and subsequently declines to baseline levels. The cluster around Xi’an Marathon reflects a similar pattern but with an additional minor peak before the event’s start and a peak in popularity at the event’s commencement.

Iconic events, including Beijing, Shanghai, and Xiamen marathons, all fall within the cluster with Xi’an Marathon as the clustering center. These events share similar trends in seasonal popularity, showing an upward trend with oscillations before the event registration, one or more small peaks before the event starts, and reaching peak popularity at the start of the event, followed by a gradual decline to normal levels after the event. The Shanghai Marathon is one of the most popular marathons in China, certified as a platinum-level marathon by the International Association of Athletics Federations. The trend chart of its seasonal popularity shows the highest peak occurring during the registration period. This phenomenon is normal for high-profile events like the Shanghai Marathon, which have strict entry limits but have far more people interested in participating, resulting in a peak of searches for the event name and surrounding information during the registration period.

## 4. Analysis and summary

### 4.1 Analysis of characteristics of cities hosting marathon races

As of May 2022, 66 marathons in China are included in the International Association of Athletics Federations (IAAF) certified marathon event system, with 38 having established a Baidu search index, signaling their current popularity within the country. These marathons, spread across 39 cities, are largely located in the eastern, central, and southwestern regions of China. This study delves into a characteristic analysis of these marathon-hosting cities, accentuating three aspects: political, economic, and tourism facets.

#### 4.1.1 Political characteristics

Political characteristics drive the appeal of marathon events in a city. Regional administrative centers, being political resource hubs, magnetize populations and influence the surrounding areas [33, 34]. Consequently, large-scale events held in these cities typically garner increased attention. As one of the most viewed sports events, the population size and the host city’s strong regional urban influence are pivotal in maintaining the popularity of marathon events.

For instance, Beijing and Shanghai are respectively the capital of China and one of the most important municipalities and mega-cities in China. Their special political status and strong economic strength have garnered significant national and global attention. As a result, the Beijing Marathon and Shanghai Marathon are among the top three most popular marathons in China. Direct-controlled municipalities and provincial capital cities like Chongqing, Xi’an, Nanjing, Guangzhou, and Chengdu have strong political attributes both in China and their respective provinces. These cities have large populations, complete infrastructure, strong economic strength, regional influence, and the marathons held in such cities also receive significant regional attention.

#### 4.1.2 Economic characteristics

A city’s economic prowess plays an instrumental role in upholding the popularity of its marathon event. A large-scale marathon event’s successful organization necessitates significant economic, human, and material resources. Cities with stronger economic standing often witness greater success and popularity for their marathon events. It is therefore not enough to heavily invest in the marathon event alone; ensuring the city’s economic vitality is equally paramount to secure the event’s success and maintain its popularity [35].

Cities such as Shenzhen, Hong Kong, Changzhou, and Wuxi stand out among the 38 cities that host the most popular marathons in China due to their strong economic vigor and overall urban influence. It is essential for these cities to maintain their economic strength while also investing in the marathon events to ensure continued success and popularity.

#### 4.1.3 Tourist characteristics

In the context of a marathon event, a city’s tourist resources are pivotal for its popularity. Tourism characteristics can significantly enhance the event’s popularity, giving a unique impression of the city and its event [36]. While professional athletes participate in the marathon event, travel motivation often drives the majority of participants and spectators.

In designing marathon events, cities typically emphasize their unique characteristics to attract participants and audiences. The aesthetics of the race course play a crucial role in crafting a first impression of the city’s landscape to participants, attendees, and media audiences. Therefore, race organizers pay close attention to designing the race course. For example, the Liupanshui Marathon, a summer marathon, features comfortable weather conditions and natural or cultural scenic spots as the main attraction. The Shanghai Marathon route highlights the famous Bund landscape, the Huangpu River bank, and the Nanjing Road Pedestrian Street. The Xiamen Marathon follows the Island Ring Road as the main route. The Beijing Marathon’s race course integrates the cultural landscape and track conditions, starting from the iconic Tiananmen Square, passing through the financial street, Xuefu Road, Zhongguancun, and other core areas of Beijing’s economy, culture, and technology, and ending at the celebration square in the center of the National Olympic Park, where participants can admire Beijing’s Olympic legacy buildings like the Bird’s Nest and Water Cube. The Xichang Qionghai Wetland Marathon and Hengshui Lake Marathon highlight natural water bodies and enable participants to appreciate the wetland environment’s natural scenery suitable for leisure and health. Most other cities’ marathon events combine the racecourse design with local tourist resources to showcase the event and the city’s landscape and characteristics to participants and media audiences.

Tourist attractions with unique characteristics and popular online buzzwords are crucial in attracting tourists and maintaining the city’s appeal, indirectly contributing to the popularity of the marathon event. All the cities hosting the 38 most popular marathons in China boast a multitude of A-level scenic spots, as depicted in Table 4.

**Table 4:** Overview of tourist resources in 38 cities hosting marathon.

Number	City	Urban keywords	Major tourist attractions	
			5A tourist attractions name	Number of 4A
1	Shanghai	Magic City, The Bund, Shanghai nightlife, Pedestrian Street, Fashion, Internationalization	Scenic spots of the Oriental Pearl Radio and TV Tower, Shanghai Wild Animal Park, Shanghai Science and Technology Museum, and Memorial Halls for the First, Second, and Fourth National Congress of the Communist Party of China.	80
2	Xiamen	Waterfront Area, Garden city, Ring Road, Gulangyu Island, Zhongshan Road	Xiamen Gulangyu Scenic Area	10
3	Guangzhou	Metropolis, Red Culture, Canton Tower, Herbal Soup, Dim Sum	Baiyun Mountain Scenic Area, Chimelong Tourist Resort,	32
4	Shenzhen	Openness, inclusiveness, innovation, vitality, technology, Guangdong-Hong Kong-Macao Greater Bay Area	Overseas Chinese Town Tourist Resort, Guanlanhu Leisure	8
5	Kunming	Spring City, Flower City, Happiness, Livability, Ancient City	Expo Park, Shilin Scenic Area	11
6	Hefei	Cultural atmosphere, transportation hub, technological innovation, urban center	Sanhe Ancient Town	26
7	Nanchang	Heroic Nanchang, Tengwang Pavilion, transportation hub, rice noodles	Tengwang Pavilion Tourist Area	11
8	Hangzhou	West Lake, Jiangnan rain, technological innovation, silk culture, Longjing tea	Xixi Wetland Tourism Area, West Lake Scenic Area	46
9	Xichang	Aerospace city, ancient civilization city, small spring city, poverty alleviation		14
10	Wuhan	Central core city, transportation hub, Hot dry noodles, Yellow Crane Tower, Yangtze River Bridge	East lake Scenic Area, Yellow Crane Tower Park	22
11	Nanjing	Ancient city of Jinling, cultural atmosphere, transportation hub, duck blood vermicelli, sci-tech innovation platform	Confucius Temple-Qinhuai River Scenic Belt Tourist Area, Zhongshan Mausoleum Scenic Area	25
12	Chengdu	Science and Technology New City, hot pot, slow living, cultural city, western central hub city	Qingcheng Mountain-Dujiangyan Tourism Area	48
13	Suzhou	Suzhou Gardens, steady economy, technology, cultural atmosphere	Suzhou Gardens, Tongli Ancient Town, Wuzhong Taihu Tourism Area, Zhouzhuang Ancient Town, Shajiabang-Yushan Shanghu Tourism Area, Jinji Lake Scenic Area	34
14	Hong Kong	International financial center, international transportation center, cultural center, economic center, shopping paradise		
15	Dongying	Yellow River estuary, petroleum, seaside	Yellow River estuary Ecotourism Area	7
16	Beijing	Imperial capital, Great Wall, Forbidden City, political center, cultural center, Olympics	Badaling-Mutianyu Great Wall Tourist Resort, Ming Tombs Scenic Area, Temple of Heaven Park, Palace Museum, Olympic Park, Yuanmingyuan Park, Prince Gong’s Mansion, Summer Palace	72
17	Changzhou	Heavy industry city, Suzhou-Wuxi-Changzhou metropolitan area, downstream of the Yangtze River economic belt	Tianmu Lake Scenic Area, Universal Dinosaur Town Leisure Tourism Area, China Spring and Autumn Yancheng Tourism Area	10
18	Liupanshui	Cool city, livable, Beipanjiang, cherry blossom, Three Pools and Three Lakes		5
19	Jilin	Ice and snow city, Songhua River, heavy industry, Manchu culture		11
20	Dalian	Livable, coastal, tourist city, cultural atmosphere, streets and lanes	Tiger Beach Ocean Park, Golden Pebble Beach Scenic Area	20
21	Xi’an	Ancient city of Chang’an, Terracotta Warriors and Horses, historical and cultural heritage, university city, national central city	Qinshihuang’s Mausoleum Museum, City Wall-Beilin Historical and Cultural Scenic Area, Big Wild Goose Pagoda and Da Tang Furong Garden Scenic Area, Huaqing Pool Scenic Area, Daming Palace Tourist Area	32
22	Yangzhou	Ancient city of Yangzhou, Grand Canal, Huaiyang cuisine, historical and cultural heritage, tourist city, livable	Slender West Lake Scenic Area	15
23	Wuxi	Taihu Lake, Suzhou-Wuxi-Changzhou, thick soy sauce, spare ribs	Lingshan Scenic Area, Yuantouzhu Scenic Area, CCTV Wuxi Film and Television Base Three Kingdoms Shuihu Scenic Area, Huishan Ancient Town	27
24	Fuzhou	City of Banyan Trees, land of blessings, fish balls, hot springs, capital city of Fujian Province	Three Lanes and Seven Alleys Scenic Area	10
25	Nanning	Garden city, cleanliness, inclusiveness	Qingxiu Mountain Tourist Area	30
26	Changsha	Stinky tofu, Yuelu Academy, Orange Island, central super city, fireworks	Yuelu Mountain and Orange Island Tourist Area, Shenyan Botanical Garden, Sun Island Scenic Area, Huaxi Qingyan Ancient Town Tourist Area	24
27	Shenyang	Chicken skewers, Erren Zhuan, historical and cultural city, industrial base	Shenyang Botanical Garden	17
28	Hengshui	Hengshui Lao Bai Gan, Jizhou, Hengshui Middle School		4
29	Taiyuan	Energy industry base, historical and cultural city, Jin merchant culture, important central city in the central region		10
30	Haerbin	Ice city, electrical industry, Harbin beer, Central Avenue, cold	Harbin Sun Island Scenic Spot	29
31	Lanzhou	Yellow River runs through, beef noodle soup, Silk Road Town, rich culture		8
32	Guiyang	Important central city in Southwest China, big data industry agglomeration area, Southwest transportation hub, summer resort	Huaxi Qingyan ancient town scenic spot	10
33	Chongqing	Mountain city, hotpot city, fog city, bridge city, cultural and creative industry	Wulong Karst Tourism Area, Youyang Taohuayuan Tourism Area, Wushan Xiaosanxia-Small Three Gorges, Yunyang Longgang Scenic Area, Pengshui County Ayi River Scenic Area, Jiangjin Simianshan Scenic Area, Dazu Rock Carvings Scenic Area, Wan Sheng Economic Development Zone Black Mountain Valley Scenic Area, Nanchuan Jinfo Mountain, Qianjiang Zhaoshui Scenic Area	111
34	Xuzhou	Historical and cultural city, transportation hub, Huaihai economic zone, hometown of emperors	Yunlong Lake Scenic Area	10
35	Yichang	Three Gorges of the Yangtze River, livable, water and land transportation hub, Yangtze River middle reaches urban agglomeration	Three Gorges Dam-Qu Yuan’s Hometown Tourist Area, Three Gorges Folk Customs Scenic Area, Changyang Qingjiang Gallery Scenic Area	20
36	Zhengzhou, Kaifeng	Zhengzhou: National key hub city, central core city, historical and cultural city, Hui noodles; Kaifeng: Qingming Riverside Park, eight dynasties ancient capital, historical and cultural city, origin of Henan Opera	Qingming Riverside Landscape Garden	Zhengzhou:10 Kaifeng:5
37	Qingyuan	Guangfu culture, Guangzhou metropolitan area cities	Lianzhou Underground River Tourism Scenic Area	16
38	Shaoxing	Ancient city of Shaoxing, small bridges over flowing waters, yellow wine, historical and cultural relics.	Lu Xun’s Hometown Shen Garden Scenic Area	10

### 4.2 Marathon event popularity trend analysis

Popularity trend heat maps of major marathon events display both similarities and divergences. A shared characteristic is the presence of multiple popularity peaks, with the highest typically coinciding with the race commencement. Analysis shows that key time markers, such as event initiation, registration, lottery result publication, and the race start, often align with peaks in event popularity. However, the variation in the number of peaks, the considerable differences in popularity values for the same event, and the disparate time intervals between peaks highlight discrepancies in the frequency and timing of promotional activities and key social events during each event cycle, thereby eliciting different levels of public attention.

The popularity trends of China’s three most representative marathons remain relatively stable due to their superior maturity, the breadth of supporting events, and frequent event releases. Yet, each event possesses distinctive features. For instance, the 2015 season’s Shanghai Marathon experienced a minor popularity peak at the start of registration on September 2nd. The most significant peak occurred on September 21st, corresponding with the lottery result publication, as the Shanghai Marathon introduced a lottery system to ensure race orderliness. Post September 21st, popularity rapidly declined, gradually increasing again on October 2nd and reaching another peak on the race day, October 8th.

Some marathon events witness the emergence of a new minor popularity peak due to the rising number of participants and constraints of racecourse conditions and participant limits. To mitigate participant influx, independent supporting events such as half marathons and 5km/10km health runs are introduced. For example, the Shanghai Marathon series incorporated the Shanghai Half Marathon in 2015, the 10km Elite Race in 2016, the Shanghai International Women’s Half Marathon in 2019, and the Suzhou River Half Marathon in 2023, thereby forming an extensive event system. Similarly, the Xiamen International Marathon Half Marathon was independently held in Haicang District in 2016 and was renamed the Xiamen (Haicang) International Half Marathon. In 2020, the Xiamen Huan Dong Half Marathon was added as a derivative event, thereby enhancing the Xiamen Marathon series.

### 4.3 Path analysis of urban marathon events driving urban development

#### 4.3.1 Driving the urban economy

Marathon events constitute a highly successful category of sports tourism in China, often yielding substantial economic benefits for the host city [30]. These benefits can be divided into two primary forms: direct benefits to the event organizing committee and comprehensive economic benefits to the city.

Firstly, as a symbol of sports tourism and the sport attracting the highest number of participants, marathon events often draw tens of thousands of runners. Registration fees represent a significant income for event organizers. Given the broad audience and high exposure marathon events attract, they can draw numerous sponsors and partners through commercial operations, with sponsorship income being a crucial component of direct benefits. However, organizing a large-scale single sport necessitates substantial operating funds to ensure the event’s success. Direct benefits often offset operating costs or may require additional financial subsidies. Currently, only a few marathon event organizers can achieve a balance of income and expense [37].

Secondly, marathon events have notable comprehensive economic benefits for the host city. As a symbol of sports tourism, a successful and highly popular marathon event, closely integrated with the host city’s tourist attractions, unique landscapes, and cultural characteristics, often attracts tens of thousands of participants and accompanying individuals driven by competition and travel. Their consumption of transportation, catering, accommodation, tourism, and shopping directly infiltrates various economic sectors of the host city, directly boosting the city’s GDP growth [38]. For instance, the 2021 Xiamen Marathon in China generated direct economic benefits of approximately RMB 119 million and comprehensive economic benefits of approximately RMB 301 million. The historical number of participants in the Xiamen Marathon exceeded 720,000, and the comprehensive economic benefits accumulated to RMB 4.49 billion, with a brand value of RMB 2.293 billion. Furthermore, the large influx of people in a short period of time continually promotes the discovery and creation of new growth points in the market economy. Additionally, there is the possibility of participants staying in the host city for work, life, or tourism after participating in the race or sightseeing, and even the possibility of investment intentions and behavior, which become the potential economic impact of the marathon event on the city.

#### 4.3.2 Enhancing the city image

Marathon events are becoming one of the most prominent events in each host city, substantially contributing to the city’s branding and influence. The city image is an amalgamation of a city’s natural landscape, cultural ambiance, and resident quality, providing the primary sensory foundation for forming basic judgments and impressions of the city. Hosting a city marathon event requires the collaborative effort of various fields within the city, not solely the effort of the event organizing committee. For instance, the race track’s landscape design significantly impacts the overall visual effect, and the supply of water, food, and other services during the event closely relates to the city’s image.

#### 4.3.3 Promoting urban supporting facilities

To meet the requirements of hosting a marathon event, host cities partially construct or refurbish urban tracks, purposefully design or embellish the visible landscapes around the track to ensure a positive first impression for participants. This preserves and increases the possibility of second participation or travel, significantly improving the overall city tourism landscape.

During the event, a large number of participants and accompanying tourists suddenly flood into the city, posing a significant challenge to the city’s transportation, hotel, and catering industries. To maximize the participants’ sensory experience during the event, higher infrastructure and surrounding service requirements are proposed, promoting the upgrading of urban supporting facilities and service improvement to meet the needs of higher standards for urban events [39]. Lay a certain foundation for the construction of smart cities [40].

Issues such as social order, fire safety, and medical care become increasingly critical during the event. The recurrent nature of marathon events enhances a city’s preparedness for large-scale emergencies over time. Unlike single-occurrence events, marathons are cyclical, thus, infrastructure and ancillary services that evolve in response to the event persist, playing a significant role year after year. They gradually become an integral part of the city’s daily life, expanding the city’s activity and tourism spaces, thereby improving the urban spatial configuration and auxiliary services.

### 4.4 Enhancement path for city development driven by marathon events

Currently, successful host cities of marathons exhibit distinct political, economic, and tourism characteristics, offering valuable guidance for ordinary cities aiming to host marathons and leverage the influence of these events to drive urban development. A successful marathon event can attract heightened attention, generate substantial economic benefits, and foster more coherent urban spatial structures and supporting infrastructure within the host city. The integration of events and urban development mutually reinforce each other, necessitating organizers to dedicate ample attention and investment to various aspects of the event.

First and foremost, it is crucial to elevate political attention and increase economic investment by harnessing the event’s economic attributes to drive comprehensive economic benefits and enhance the city’s visibility. As a mega-event, marathons require the mobilization of numerous social resources, encompassing transportation, hotels, catering, volunteers, medical services, and more. This entails increased political efforts and the allocation of policy resources from government departments, ultimately improving governance efficiency, administrative execution, and the allocation of social resources. The economic attributes of marathon sports tourism should be fully harnessed by allocating additional funds to event security and promotion, with the aim of enhancing participants’ experience and sensory perception, thereby fostering stronger connections between participants and the event as well as the host city. Highlighting inspiring stories and noteworthy incidents occurring during the event can effectively attract media and public attention, thus increasing the overall exposure of the city.

Secondly, it is essential to leverage the event’s tourism attributes, integrate urban tourism resources, and enhance the urban tourism spatial structure to showcase the city’s landscapes and cultural characteristics to participants and spectators. As a prominent sports tourism event, marathons possess a strong tourism attribute, with a high degree of overlap between participants’ tourism behaviors during the event and their regular tourism activities. Therefore, host cities should fully utilize their tourism resources, identify and showcase their unique urban characteristics, and integrate available tourism resources with the marathon event, enabling participants to fully immerse themselves in the city’s cultural charm and scenic beauty. In cases where a city’s distinctive features are not readily apparent or are dispersed, proactive efforts should be made to position and showcase desired urban characteristics, capitalizing on the city’s advantages to create new urban features that can be highlighted to the world through the platform of the marathon event. Examples of recent trends include rapeseed flower marathons, tulou marathons, and mountain marathons.

Lastly, establishing partnerships with well-known marathon events can enhance the regional influence and participation of the event, thereby further expanding the city’s overall influence and regional reputation. With the proliferation of marathon events, some cities may face challenges in independently creating influential marathons due to limited influence. In such cases, cities can consider collaborating with neighboring cities to develop distinctive events such as marathon relays or team races. Another strategy is to seek partnerships with renowned marathon events, such as elevating local marathon events to the level of qualification races for top-tier marathons or securing a certain number of direct entry slots through lotteries or rewards for top-ranking athletes. These approaches establish connections between cities and events, enabling the development of local marathon events by leveraging the influence and experiences gained from high-level events and host cities.

## 5. Conclusion

We utilized machine learning algorithms to mine the Baidu search index of marathon events, and combined this with city characteristics for analysis. Our goal was to identify the features and pathways that city marathons contribute to urban development. Clustering results based on the Baidu search index allowed us to categorize marathon events with similar trends in popularity, as well as to identify representative seasonal popularity trends for these events. The cities hosting different categories of events exhibited distinctive political, economic, and tourism features. By analyzing the correlation between these features and the representative seasonal popularity trends, we were able to identify characteristics of the host cities for these representative events. Interestingly, we found many cities with characteristics similar or identical to those in our study, suggesting that the successful experiences of urban development driven by marathons may offer new insights for the development of these cities.

Extracting city features can enable cities with similar characteristics to rapidly explore sports tourism pathways suitable for their own urban development. Aspects such as city policies towards events, infrastructure, human and material resources are crucial guarantees for the successful hosting of events. The tourism characteristics of a city form the essential foundation for the development of sports tourism and serve as one of the main motivations for participation. Cities with tourism infrastructure can compare their political, economic, and tourism perspectives with different city categories and find benchmark city categories.

Maintaining event stimulation during the season is vital for sustaining event popularity. Every peak in popularity during the season represents an event occurrence at that time point. Analyzing the representative event popularity changes can help host cities understand the stimulating events that can be used at different stages of the event, as well as the frequency of appropriate event stimulation. For instance, when the event matures to a certain stage, it can be divided into several sub-events to achieve continuous stimulation and maintain long-term event popularity.

The successful experience of city development driven by marathons is an important reference for the development of other cities. We have summarized the current pathways of city development driven by city marathons, analyzed the deficiencies in conjunction with important city characteristics, and further proposed enhancement pathways for city development driven by city marathons. Among these, combining the city’s characteristics to co-create joint events with mature host cities is an excellent enhancement pathway. There are still some deficiencies in this study, such as the lack of research on the role of city marathons in city development after the end of the pandemic, given that our study data only goes up to May 3, 2022. In the future, we plan to update and complete our research on this topic.

## Supporting information

S1 Dataset (XLSX)
